# Inhibitory effect and mechanism of *Tagetes erecta* L. fungicide on *Fusarium oxysporum* f. sp. *niveum*

**DOI:** 10.1038/s41598-017-14937-1

**Published:** 2017-10-31

**Authors:** Ruochen Du, Jiandong Liu, Panpan Sun, Hongquan Li, Jinsheng Wang

**Affiliations:** 1 College of Animal Science and Veterinary Medicine, Shanxi Agriculture University, Taigu, Shanxi 030801 P.R. China; 2 College of Life Science, Shanxi Agriculture University, Taigu, Shanxi 030801 P.R. China

## Abstract

Botanical fungicides comprise attractive alternatives to chemical fungicides because of their environmental compatibility. Flavonoids extracted from *Tagetes erecta* L. have an inhibitory effect on fusarium wilt in watermelons caused by *Fusarium oxysporum* f. sp. *niveum* (FON). In this study, we synthesized one of these flavonoids, 2,5-dicyclopentylidene cyclopentanone (*Tagetes erecta* L. fungicide (TEF)) and assessed its activity against FON. *In vitro*, TEF inhibited FON growth and killed FON cells directly. TEF also affected FON cell physiology and mycelial structure. In watermelon plants with fusarium wilt, TEF protected the leaf cell structure and improved the germination rate of infected seeds while increasing overall plant resistance. A TEF-resistant mutant (FONM) was created by chemical mutagenesis. FON and FONM were analysed using iTRAQ and RNA-Seq, which identified 422 differentially expressed proteins and 7817 differentially expressed mRNAs in the proteome and transcriptome, respectively. The FONM mutations caused changes in the cell membrane and cell wall, which may constitute the site of action of TEF. Together, these results demonstrate that TEF could effectively control the watermelon fusarium wilt caused by FON, possibly through the inhibition of sterol biosynthesis. The data presented here suggest that TEF represents a new potential botanical anti-fungal drug.

## Introduction

Fusarium wilt of watermelon is caused by *Fusarium oxysporum* f. sp. *niveum* (FON). Worldwide, the loss of watermelons owing to FON infection has been increasing annually. As the process of breeding for FON-resistant cultivars can be challenging^1^, chemical pesticides such as carbendazim, myclobutanil, and diniconazole have traditionally been used to control FON infections^2^. However, these drugs have high potential risks^3^. Furthermore, with continued use of these pesticides, FON has evolved to become pesticide-resistant. Alternatively, botanical fungicides have long been considered desirable substitutes to chemical fungicides because of their lower threat to the environment and to human health^4^. Currently, the development of botanical fungicides is mainly focused on plant extracts such as alkaloids, flavonoids, and essential oils^5,6^. It has been shown that plant extracts possess antifungal activity^7^. To date, there have been no reports on the botanical fungicides to treat watermelon fusarium wilt caused by the FON pathogen.

*Tagetes erecta* L. is an annual ornamental plant. It has been shown that extracts of the marigold flower have antibacterial activity^8^. In addition, in our previous study we demonstrated that the extracts prepared from the marigold root had inhibitory effects on a variety of common plant pathogens^9^. An analysis of the different chemical components found in the root extract revealed that the flavonoids exerted an obvious inhibitory effect on FON^10^. However, the extraction of these flavonoids directly from marigold roots for use on an agricultural scale was impractical. To overcome this limitation, we have developed and patented a method to chemically synthesize the active substance (referred to as TEF; Patent No: ZL200810055370.X, China). The aim of the present study is to assess TEF’s antifungal effect on FON, its protective effect against fusarium wilt and the mechanism of action involved.

## Results

### Confirmation of TEF by gas chromatography/mass spectrometry (GC/MS)

**Figure 1:**
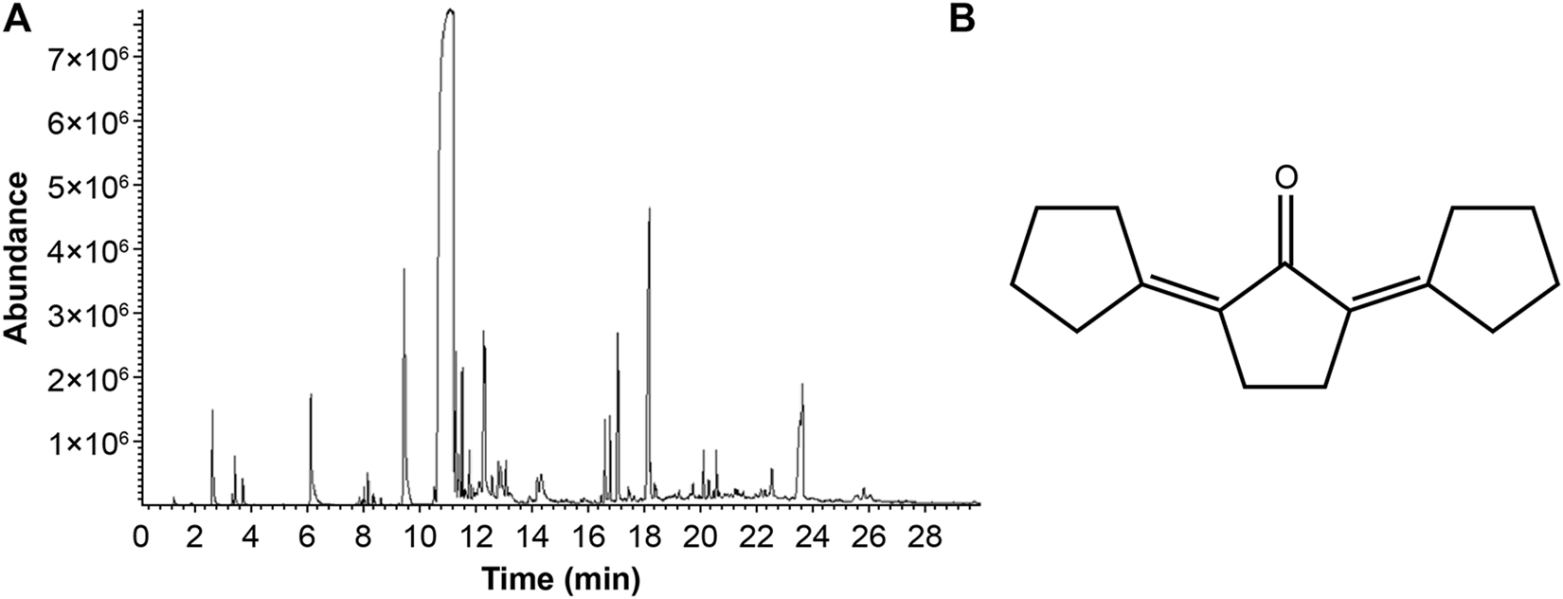
GC/MS profile and chemical formula of TEF. (**A**) GC/MS profile of TEF, showing the maximum absorption peak at 11–12 min. (**B**) Chemical structure of TEF, 2,5-dicyclopentylidene cyclopentanone. The purity is 97.2%.

The maximum GC/MS absorption peak occurred at 11–12 min (Fig. 1A). Based on the VG11-250 data retrieval system, it was confirmed that the synthesized material is 2,5-dicyclopentylidene cyclopentanone with a purity of 97.2%.

### Effect of TEF on FON

**Figure 2:**
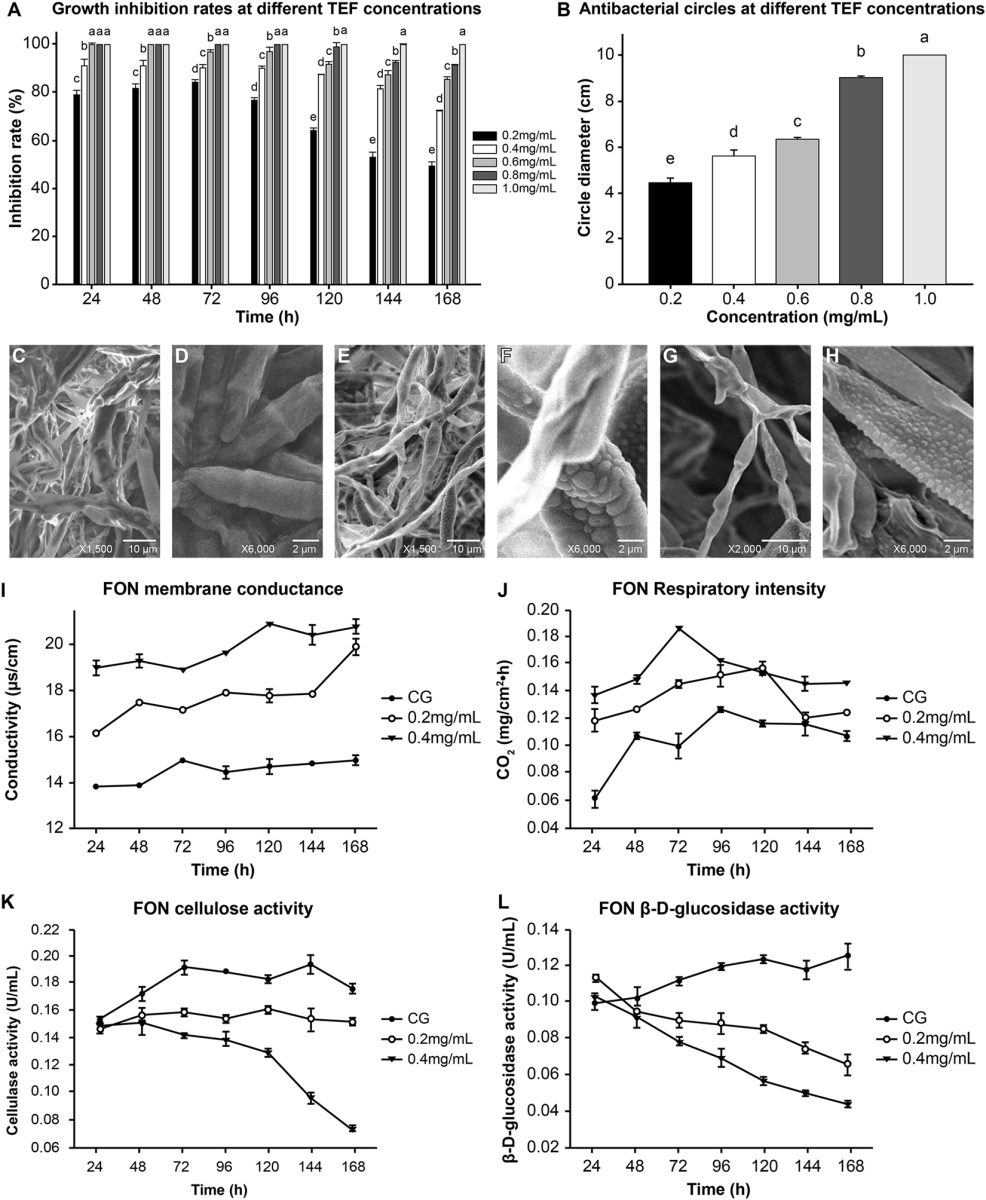
Effect of TEF on FON *in vitro*. (**A**) Growth inhibition rates over 24–168 h. (**B**) Anti-fungal zone of inhibition at 168 h. (**C–H**) Scanning electron microscopy image of FON mycelium in the control group, 1500× (**C**) or 6000× (**D**) magnification. FON mycelium in the 0.6 mg/mL TEF treatment group, 1500× (**E**) or 6000× (**F**) magnification. FON mycelium in the 0.8 mg/mL TEF treatment group, 2000× (**G**) or 6000× (**H**) magnification. (**I**) Changes in membrane conductance of FON. (**J**) Changes in respiratory intensity of FON. (**K**) Changes in cellulase activity of FON. (**L**) Changes in β-D-glucosidase activity of FON. CG: control group with no TEF. Means followed by the same letter are not significantly different at the *P* < 0.05 level.

TEF had an obvious inhibitory effect on FON growth (Fig. 2A). The inhibition rate reached 100% at a concentration of 1.0 mg/mL. The antifungal zone of inhibition (Fig. 2B) increased in size as the FON concentration increased. All FON were killed at 1.0 mg/mL of TEF. The EC_50_ value at 168 h was 0.2021 mg/mL (95% confidence intervals: 0.1919–0.2123 mg/mL).

Mycelial growth in the control group (Fig. 2C,D) was evenly distributed, with a smooth surface, as shown by scanning electron microscopy. Conversely, in the 0.6 mg/mL TEF treatment group (Fig. 2E,F), the mycelia were deformed and irregular in shape with swellings on the surface. Mycelia were less frequently observed and the expansion site was more severely deformed in the 0.8 mg/mL TEF treatment group than that in the 0.6 mg/mL group (Fig. 2G,H).

The FON membrane conductance increased with increasing concentration of TEF (Fig. 2I). The respiratory intensity in each treatment group increased initially and then decreased (Fig. 2J). As the TEF concentration increased, the overall level of respiratory intensity increased. Overall, cellulase (Fig. 2K) and β-D-glucosidase (Fig. 2L) activity decreased with increasing concentrations of TEF.

### Effect of TEF on fusarium wilt

**Figure 3:**
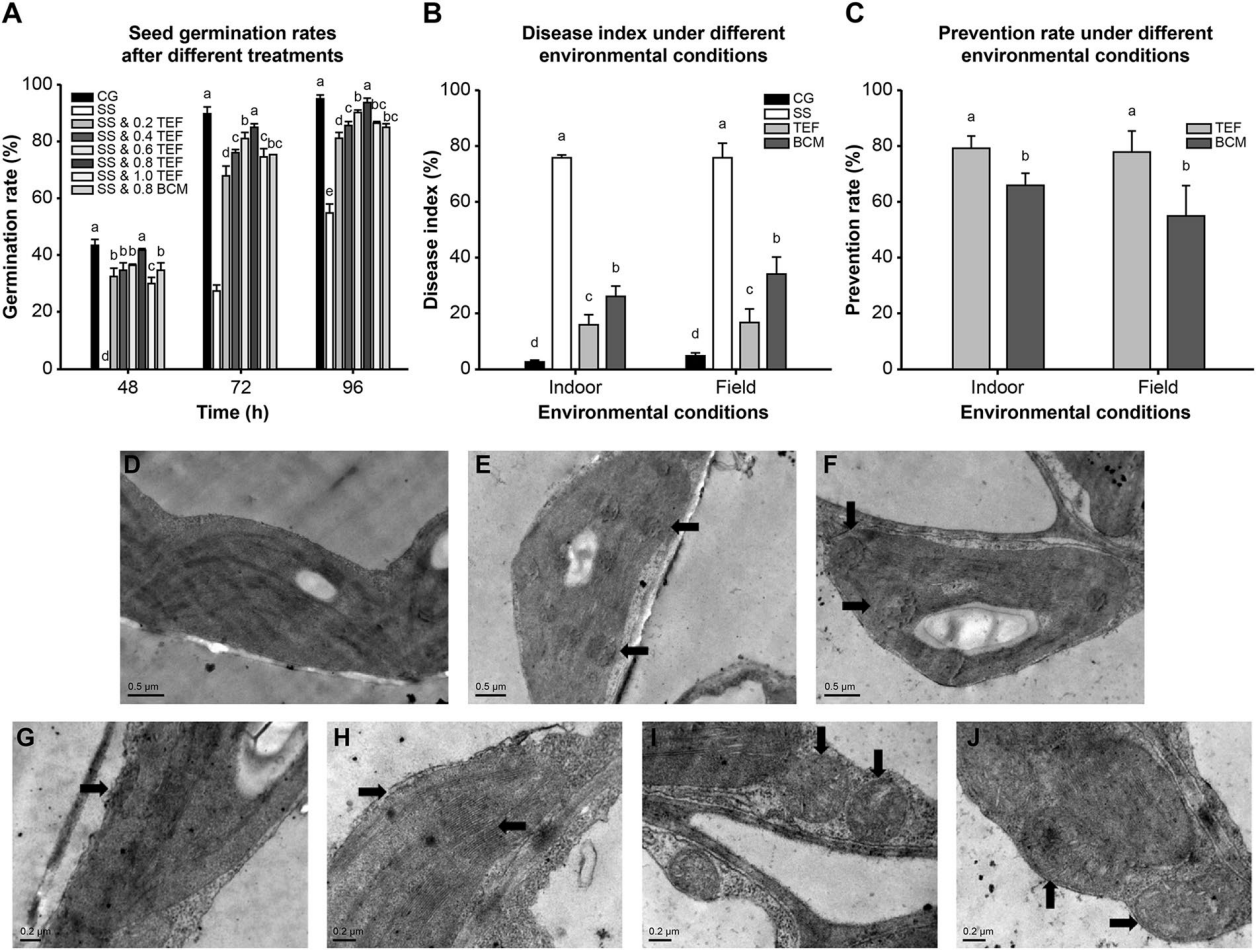
Effect of TEF on fusarium wilt. (**A**) Seed germination rates at 48, 72, and 96 h. (**B**) Seedling disease index. (**C**) Prevention rates of drugs. (**D**) Leaf tissues of the control group. (**E**–**J**) Transmission electron microscopy images of osmiophilic granules from the SS (**E**) and the SS and TEF (**F**) treatment groups. Chloroplast from the SS (**G**) and the SS and TEF (**H**) treatment groups. Mitochondria from the SS (**I**) and the SS and TEF (**J**) treatment groups. CG: control group with no drugs or SS. SS: SS treatment group; SS & TEF: SS and different concentrations of TEF treatment groups; SS & BCM: SS and 0.8 mg/mL BCM treatment group; TEF: SS and 0.8 mg/mL TEF treatment group: BCM: SS and 0.8 mg/mL BCM treatment group. Means followed by the same letter are not significantly different at the *P* < 0.05 level.

Watermelon seed germination rates, assessed at 48, 72, and 96 h, were significantly reduced in the presence of FON spore suspension (SS) (*P* < 0.05), whereas this was reversed by TEF treatment in a dose-dependent manner with a considerable effect being observed even at the lowest TEF dose of 0.2 mg/mL (Fig. 3A). The maximal effect was obtained in the 0.8 mg/mL TEF treatment group. Conversely, the effect of 0.8 mg/mL carbendazim (BCM) was significantly lower than that of 0.8 mg/mL TEF (*P* < 0.05).

TEF treatment significantly reduced the disease index in both the indoor and field settings to virtually the same extent (*P* < 0.05). BCM treatment also reduced the disease indices but appeared to have stronger effects indoors compared to that in the field environment. The TEF treatment was significantly more effective than BCM treatment in both locations (*P* < 0.05) (Fig. 3B,C).

The structure of leaves in the control group was normal and the internal organelles were clearly intact, as shown by transmission electron microscopy (Fig. 3D). The number of osmiophilic granules was found to be increased in the SS treatment group (Fig. 3E) and a clear disruption of organelle membrane architecture was observed. Thylakoid lamellae were reduced in number and unevenly distributed (Fig. 3G). In addition, the number of mitochondria was increased in the SS group and they were smaller than normal, adopting an oval, spherical shape (Fig. 3I). In the TEF treatment group, the number of osmiophilic granules was reduced as compared to that in the SS group (Fig. 3F). In addition, compared to the SS-treated group, the degree of chloroplast membrane damage was lower, with the grana-thylakoid lamellae arranged appropriately, and the number of chloroplasts was increased (Fig. 3H). The number of mitochondria was also increased in the TEF group with a larger mitochondrial volume compared to that observed in the SS treatment group (Fig. 3J).

**Figure 4:**
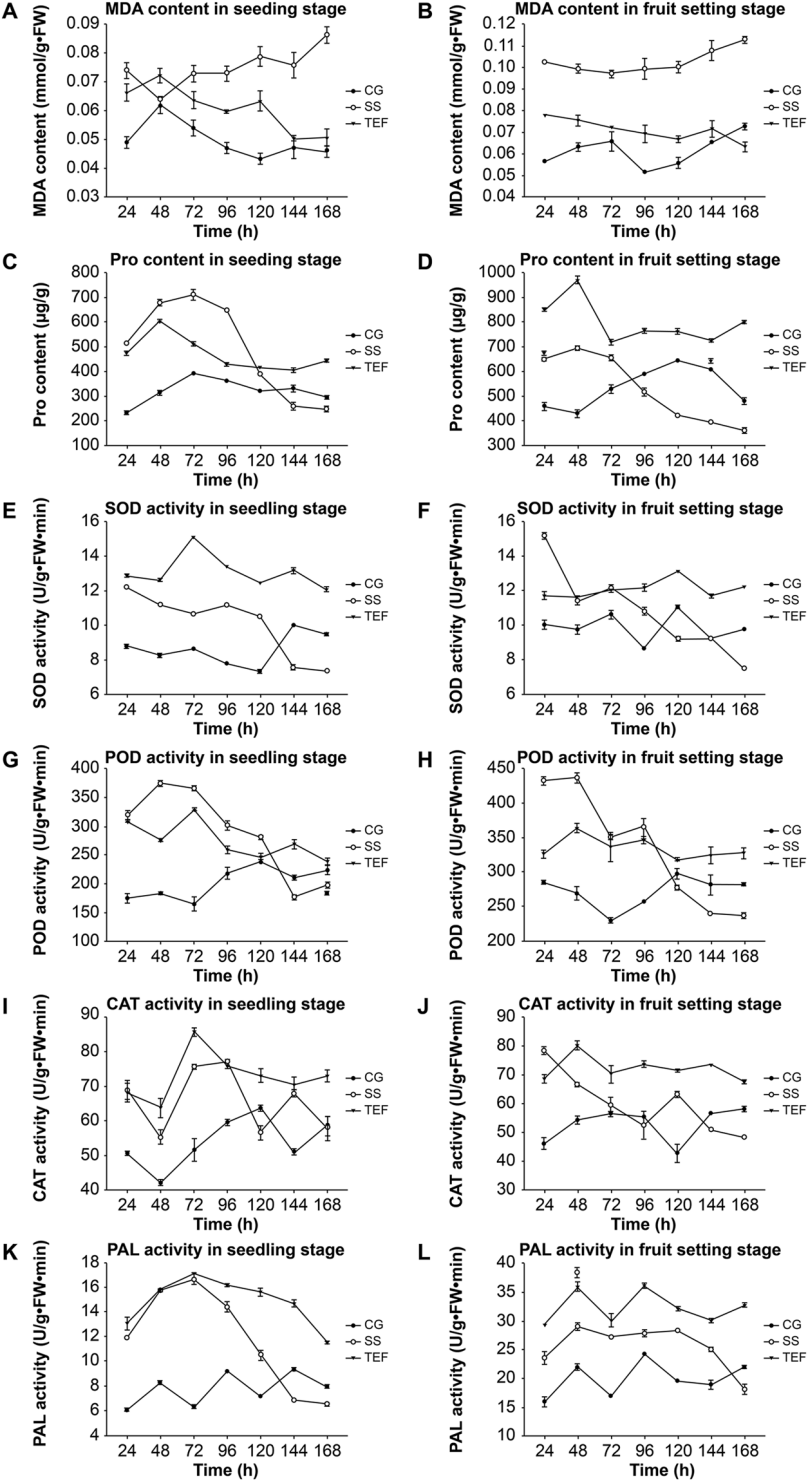
MDA and Pro content, SOD, POD, CAT, and PAL activities in leaves. MDA content at the (**A**) seedling and the (**B**) fruit blossoming stage. Pro content at the (**C**) seedling and the (**D**) fruit blossoming stage. SOD activity at the (**E**) seedling and the (**F**) fruit blossoming stage. POD activity at the (**G**) seedling and the (**H**) fruit blossoming stage. CAT activity at the (**I**) seedling and the (**J**) fruit blossoming stage. PAL activity at the (**K**) seedling and the (**L**) fruit blossoming stage. CG: control group; SS: SS treatment group; TEF: SS and 0.8 mg/mL TEF treatment group.

Compared to the SS treatment group, TEF treatment markedly reduced the malondialdehyde (MDA) content at the seedling stage and the fruit blossoming stage (Fig. 4A,B). Conversely, upon TEF treatment, the proline (Pro) content was always maintained at higher levels than those in the control at both stages (Fig. 4C,D). Superoxide dismutase (SOD) activity in the TEF treatment group was maintained at high levels and was always higher than that in the control group, whereas SOD activity in the SS treatment group was decreased to levels lower than those in the control group (Fig. 4E,F). Changes of peroxidase (POD) activity were similar to SOD except that SS treatment was high initially (Fig. 4G,H). The overall level of catalase (CAT) activity from high to low was TEF treatment group, SS treatment group and control group both in two stages (Fig. 4I,J). TEF treatment group had the hignest l-pheynlalanin ammonialyase (PAL) activity, followed by SS treatment group and control group at both stages (Fig. 4K,L).

### Colony morphology of wild-type and mutant FON strains

**Figure 5:**
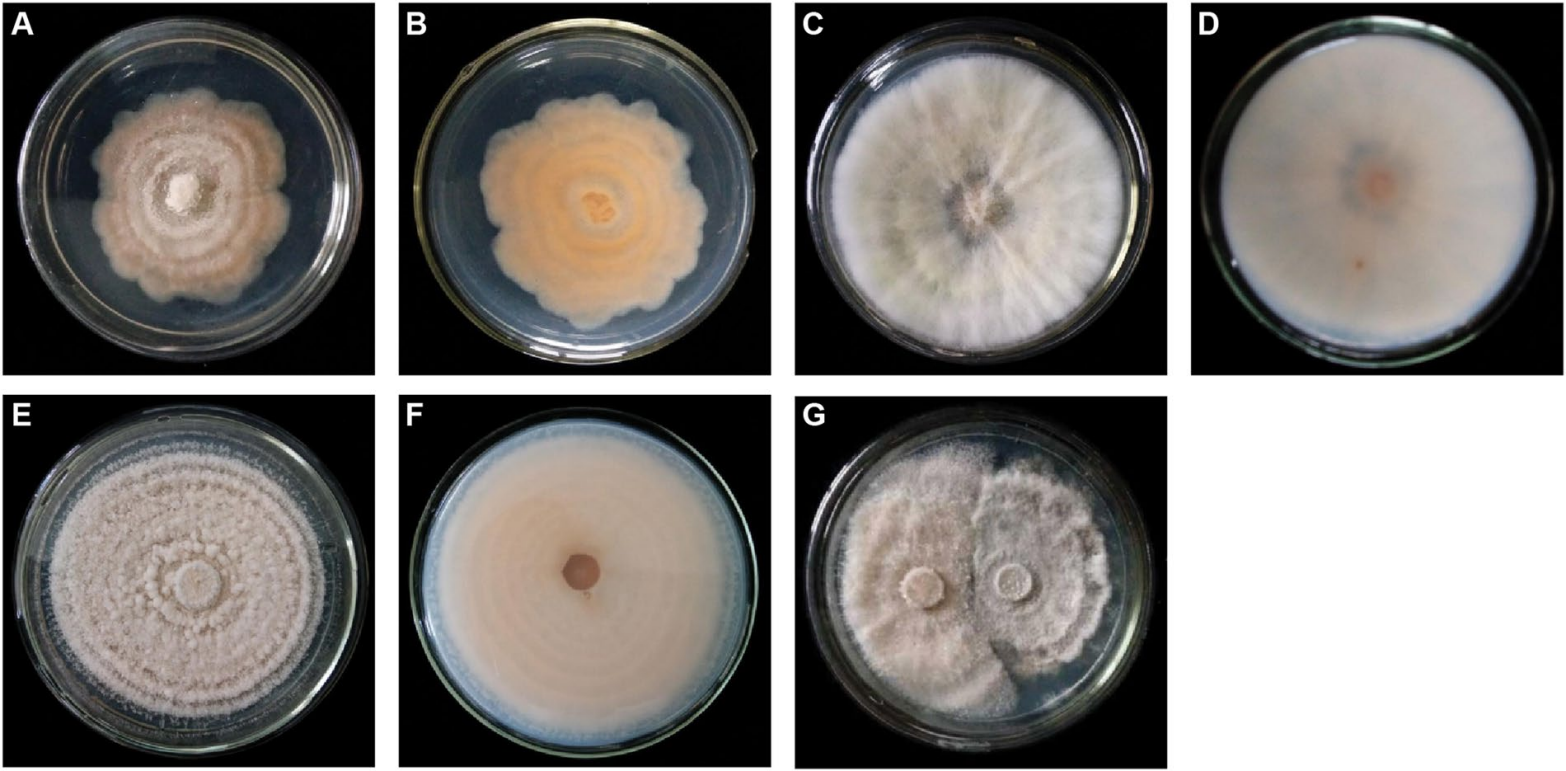
Colony morphology of FON and FONM. Positive (**A**) and negative (**B**) side of FON on PDA solid medium. Positive (**C**) and negative (**D**) side of FONM on PDA solid medium. Positive (**E**) and negative side of FONM on 0.8 mg/mL TEF on PDA solid medium. (**G**) Plate confrontation test between FON (right) and FONM (left) on PDA solid medium.

A FON mutant strain (FONM) that was resistant to TEF was obtained by mutagenesis. On solid potato dextrose agar (PDA) medium, FON grew slowly (Fig. 5A,B). In contrast, FONM grew rapidly on solid PDA medium (Fig. 5C,D), and the morphology, texture, and colour of the colony were obviously different from those of FON. After culturing of FONM on 0.8 mg/mL TEF solid PDA medium (Fig. 5E,F), the characteristics of FONM changed: the growth rate decreased and the colony adopted a yellow colour; the radial texture of the colony was attenuated; and a ring texture appeared. In a plate confrontation test (Fig. 5G), FONM could suppress the growth of FON.

### Proteomics and transcriptional analysis

**Figure 6:**
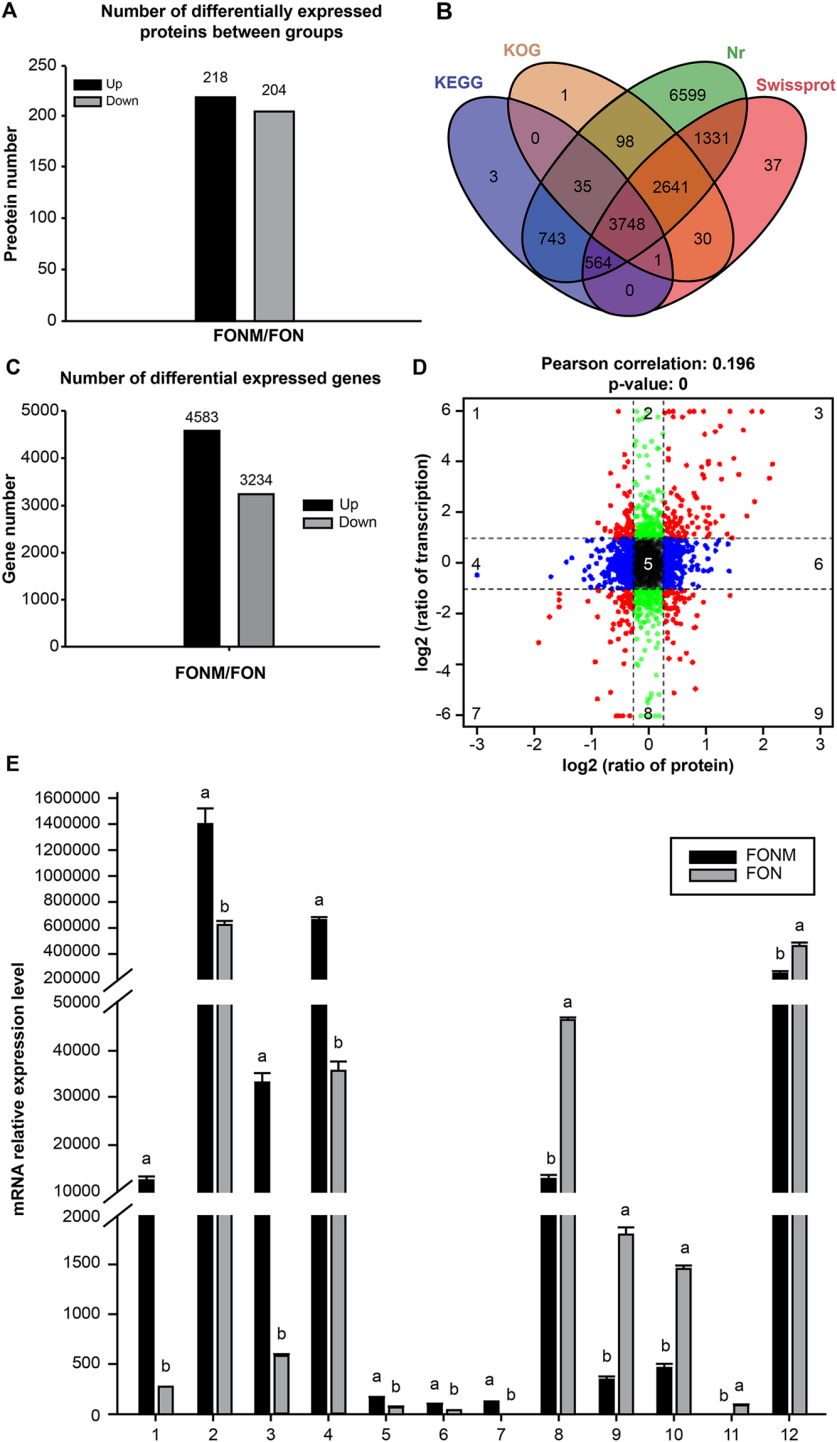
Results of proteomics and transcriptional analyses. (**A**) Number of differentially expressed proteins between groups. (**B**) Venn diagram of Unigene annotations. (**C**) Number of differentially expressed genes. (**D**) Nine quadrant chart of mRNA expression ratios and protein expression ratios [Red dots, significant changes in both protein and mRNA; blue dots, significant changes in protein only; green dots, significant changes in mRNA only]. (**E**) mRNA expression levels of key differentially expressed dots. The numbers are successively related to the key dots displayed in Table 1. Means followed by the same letter are not significantly different at the *P* < 0.05 level.

The proteomics sequencing results are shown in Supplementary Table S1. Of the 409,529 detected spectra, 86,157 were considered unique. There were 422 differentially expressed proteins between groups. Among them, 218 were up-regulated and 204 were down-regulated (Fig. 6A). Under gene ontology (GO) analysis, the most enriched GO terms were ‘metabolic’, ‘cell’, and ‘catalytic activity’ (Supplementary Fig. S1). Under Kyoto Encyclopaedia of Genes and Genomes (KEGG) analysis, the most enriched pathway was ‘carbon metabolism’, followed by ‘biosynthesis of amino acids’ (Supplementary Fig. S1).

The dataset of RNA-Seq was deposited in the NCBI database under accession number SRA541923. The results of transcriptional sequencing and the *de novo* assembly are shown in Supplementary Table S2. A total of 15,831 of the 20,889 unigenes were annotated (Fig. 6B). Among the 7817 differentially expressed genes, 4583 were up-regulated and 3234 were down-regulated (Fig. 6C). Under GO analysis, the most enriched GO terms were ‘metabolic’, ‘cell’, and ‘catalytic activity’ (Supplementary Fig. S2). Under KEGG analysis, the most enriched pathway was ‘biosynthesis of amino acids’, followed by ‘carbon metabolism’ (Supplementary Fig. S2).

**Figure 7:**
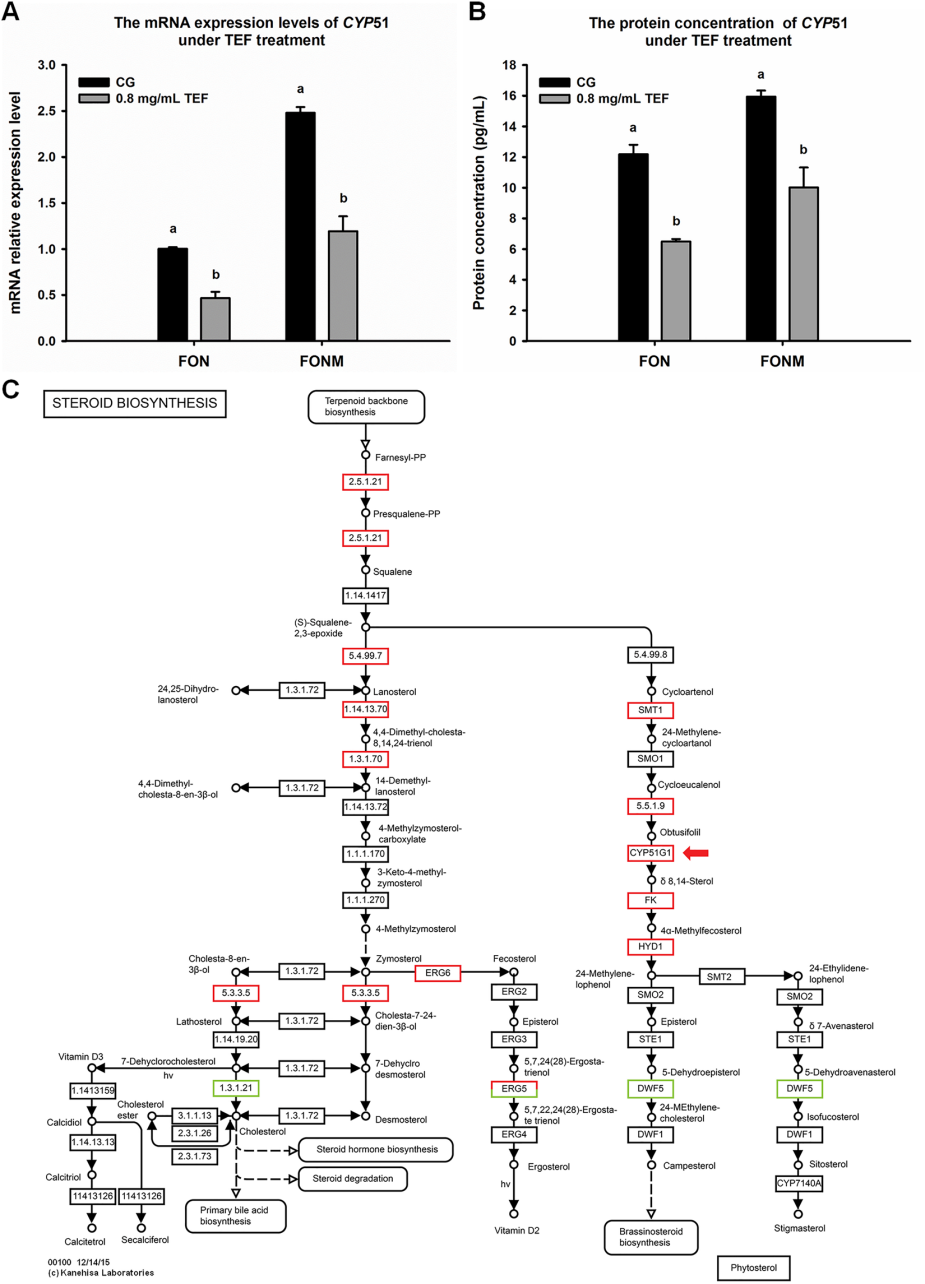
mRNA expression level, protein expression level and enrichment pathway map of *CYP*51. (**A**) mRNA expression level of *CYP*51 under 0.8 mg/mL TEF treatment. (**B**) Protein expression level of CYP51 under 0.8 mg/mL TEF treatment.CG: control group with no drugs. Means followed by the same letter are not significantly different at the *P* < 0.05 level. (**C**) Enrichment pathway map of CYP51. Map was downloaded from the KEGG server with our data mapping to the pathway (http://www.kegg.jp/kegg). Significant changes in expression are color-coded: red, up-regulated; green, down-regulated^42^. CYP51 is marked with red arrows.

The results of the association analysis revealed there is a significant relationship between protein and mRNA abundance (Fig. 6D). There were 103 and 67 dots in quadrants 3 and 7, respectively, where the changes in protein expression and gene expression levels were most consistent. The ten most differentially expressed dots in these two quadrants are shown in Supplementary Table S3, and the key differentially expressed dots based on functional annotation are shown in Table 1. The mRNA expression levels of these key dots were also analysed by quantitative reverse transcription-polymerase chain reaction (qRT-PCR; Fig. 6E). After 0.8 mg/mL TEF treatment, the RNA and protein levels of *CYP*51 in FON and FONM were significantly decreased (*P* < 0.05), but the content in FONM was always higher than that of FON (Fig. 7A,B). The pathway enrichment analysis of *CYP*51 revealed that differential expression was related to the sterol biosynthesis pathway (Fig. 7C).

**Table 1:** Key dots.

No.	Protein Acc.	FC (protein)	FC (RNA)	Description	Organism Species	Function
1	KPA37403.1	1.25	5.42	Membrane primary amine oxidase	*Fusarium langsethiae*	Membrane; oxidoreductase activity, acting on the CH-NH_2_ group of donors
2	KPA45232.1	0.80	1.17	Cell wall protein	*Fusarium langsethiae*	Cell wall; intrinsic component of cell wall
3	KPA39795.1	1.66	5.25	Membrane primary amine oxidase	*Fusarium langsethiae*	Membrane; oxidoreductase activity, acting on the CH-NH_2_ group of donors
4	CCT67575.1	1.49	4.15	Probable DUF895 domain membrane	*Fusarium fujikuroi*	Membrane; intrinsic component of membrane
5	EXA53151.1	0.33	1.29	Eburicol 14-alpha-demethylase	*Fusarium oxysporum*	Membrane; synthesize precursors of ergosterol
6	XP_964698.1	0.47	1.51	Anchored cell wall protein 4	*Neurospora crassa*	Cell wall; intrinsic component of cell wall
7	KIL86238.1	0.35	7.02	Multidrug resistance protein	*Fusarium avenaceum*	Membrane; intrinsic component of membrane;active transmembrane transporter activity
8	KPA44397.1	−0.60	−2.38	Beta-glucosidase b	*Fusarium langsethiae*	Carbohydrate metabolism; cellulose metabolic process
9	EXA36265.1	−0.46	−2.24	Beta-glucosidase	*Fusarium oxysporum*	Carbohydrate metabolism; cellulose metabolic process
10	KPA46791.1	−0.34	−1.57	Cytochrome C oxidase Assembly protein subunit 17	*Fusarium langsethiae*	Energy metabolism; intracellular part; respiratory chain complex IV assembly
11	YP_001249308.1	−0.45	−6.96	Cytochrome C oxidase subunit 2	*Fusarium graminearum*	Energy metabolism; intracellular part; generation of precursor metabolites and energy
12	EMT63775.1	−0.46	−1.41	Cytochrome C oxidase subunit 7A	*Fusarium oxysporum*	Energy metabolism; mitochondrial part; generation of precursor metabolites and energy

## Discussion

Most botanical fungicides are still in the theoretical or conceptual stage owing to several factors including difficulty in extraction and process development^11^. Obtaining these botanical fungicides in high purity and yield by other methods has therefore constituted the main obstacle to their successful application. In the present study, we obtained a botanical fungicide, originally identified in the Tagetes root, with high yield and purity by chemical synthesis.

To understand the effect of this new fungicide on FON, we initially conducted an extensive range of dose response studies to identify the optimal concentration of FON for use as a fungicide. The results showed that FON growth was completely inhibited when the TEF concentration reached 1.0 mg/mL and at this same concentration, all FON were killed in an antifungal zone of inhibition experiment. These results indicate that TEF played a dual role in inhibiting the growth of FON and directly killing FON, and that 1.0 mg/mL was the optimal TEF concentration *in vitro*. In addition, the TEF-treated FON mycelium was found to be sparser than that of untreated FON. Swelling was also noted and this resulted in considerable deformity. As currently widely used triazole fungicides can affect the formation of the fungal cell wall, resulting in cell changes including irregular swelling and considerable thickening^12^, we concluded that TEF changes FON cell wall components. Furthermore, the measurement of electrical conductivity reflects the permeability and integrity of the membrane^13^. In the present study, increasing concentrations of TEF led to increases in membrane conductivity, suggesting that the mode of action of FON is at the membrane. This process would then lead to cell leakage and consequently suppression of FON growth. Notably, cell conductance did not change further over time, suggesting that the effect of TEF on FON was rapid and long-lasting.

We also observed that FON respiratory intensity increased with increasing concentrations of TEF, and that there was an initial increase in respiratory intensity followed by a decrease. From this, we inferred that the cause of this phenomenon was related to increased respiration in FON to produce more energy to resist the TEF-induced stress, but this adaptive mechanism gradually waned under the continuous influence of TEF. This is consistent with a report by Fugro *et al*.^14^ demonstrating that BCM can affect fungal energy metabolism and ultimately inhibit their growth. Furthermore, the activities of cellulase and β-D-glucosidase are related to the infection ability of FON^15^. The data from our study demonstrate that both cellulase and β-D-glucosidase activity decreased with time in the TEF treatment group in a dose-dependent manner. These findings indicate that TEF could potentially inactivate FON infection ability by repressing cellulase and β-D-glucosidase activities.

Watermelon fusarium wilt is a soil-borne disease caused by FON that can occur over the entire watermelon development period^16^. To understand the actual preventive effects against watermelon fusarium wilt, our research focused on the key indices during germination, seedling formation, and the fruit blossoming stage. The germination rate in the 0.8 mg/mL TEF treatment group was significantly higher than that in the other groups including the BCM treatment group. The optimal TEF concentration to protect the germination process was found to be 0.8 mg/mL. This concentration was lower than the optimal TEF concentration from the *in vitro* studies described above, possibly reflecting toxic effects on the plant itself, as has been observed for other drugs. In addition, TEF treatment significantly reduced the disease index in seedlings grown either indoors or under field conditions, with a prevention rate significantly higher than that of BCM. Notably, compared to the indoor environment, the prevention rate of TEF in the field was virtually unaltered. However, the effect of BCM was obviously decreased in the field compared to that indoors. This discrepancy may be due to a complex microbial population as well as other conditions. We inferred from this that TEF might have a wider range of antifungal activity or better adaptability than BCM.

A previous analysis by Wang *et al*.^17^ on the effects of fusaric acid on cucumber leaves indicated that the leaf cell membrane was damaged and the respiratory function of mitochondria was affected. Conversely, according to our data, TEF-treated plants exhibited minimal changes in leaf cell structure; furthermore, the structures of chloroplasts and mitochondria were relatively unchanged and appeared to be largely intact compared to that in fusarium wilt-infected plants. To further understand the specific changes in watermelon leaves, we examined various biochemical indices at both the seedling and the fruit blossoming stage. Plant MDA and Pro levels, and SOD, POD, CAT, and PAL activities are important indicators of a plant’s resistance^18,19^. Chang *et al*.^20^ studied the biological effect of arbuscular mycorrhizal fungi (AMF) on fusarium wilt in cucumbers and found that the content of Pro after AMF treatment was significantly higher than normal. Bill *et al*.^21^ used thyme oil obtained from avocado to treat the fungal infection anthracnose, and the results showed that the activities of SOD, POD, CAT, and PAL all increased after treatment. Similarly, in the present study, the above mentioned indicators in the TEF treatment group remained at a beneficial level when compared to the SS treatment group. These findings indicate that TEF is able to sustain the beneficial effect of these biochemical indices and thereby increase the stress resistance of the watermelon.

The development of a drug-resistant mutant strain is crucial in studies of the mechanism of drug action. In particular, Hu *et al*.^22^ used a mutant strain to explore the mechanism of resistance to isoprothiolane (IPT) by the rice blast fungus *Magnaporthe oryzae*, and discovered that the MGG_09793 protein was significantly and specifically induced by IPT in sensitive strains. We isolated a TEF-resistant strain in order to conduct an in-depth study of the TEF antifungal mechanism. Several previous studies have shown that fungal resistance was mainly caused by genetic mutations. For example, benzimidazole resistance is often caused by point mutations in the β-tubulin gene, which result in amino acid sequence changes in the benzimidazole binding site^23^. The present study showed that although the characteristics of FON and FONM were obviously different there was no significant difference (except the growth rate) in FONM before and after TEF treatment. Therefore, we deduced that the resistance of FONM was likely to be caused by gene mutation.

To further narrow the scope of the study, we identified differentially expressed genes that showed consistent changes at both the RNA and protein levels by correlation analysis. Compared to FON, significantly up-regulated proteins in FONM mostly comprised enzymes with catalytic activity. In addition, several cell membrane proteins were also identified. At the mRNA level, the changes focused on membrane composition and function, with the largest change being in a membrane multidrug resistance protein, the mRNA abundance of which in FON was very low (approximately 1/115 of FONM) as determined by qRT-PCR. Homologs of this protein are located at the endothelial and epithelial cell surfaces and are involved in multidrug transport, with the function of expelling drugs to the outside of cells. These multidrug resistance proteins are also involved in cytochrome P450 metabolism^13^. Based on this finding, we further screened for proteins related to the cell wall and cell membrane function. The results identified seven relevant proteins, the protein and mRNA expression of which were both up-regulated. These results indicate that FONM sustains obvious differences in the composition and function of the cell wall and cell membrane when compared with FON. Combined with the effect of TEF on the mycelium surface, we concluded that the cell wall and cell membrane may constitute the site of action of TEF.

In particular, one of the seven proteins we identified is eburicol 14-alpha-demethylase (CYP51), which is necessary for cytochrome P450 synthesis. Other studies have shown that azole drugs affect the synthesis of ergosterol precursors by inhibiting the expression of CYP51, thereby impacting the structure of the fungal membrane. The ergosterol metabolic pathway has been previously described in details^24^. In the present study, the mRNA and protein expression levels of CYP51 were significantly up-regulated in FONM, and the expression of genes in related pathways was also altered. Furthermore, the mRNA and protein levels of CYP51 in FON and FONM were decreased after 0.8 mg/mL TEF treatment, although the content in FONM was always higher than that of FON. Thus, TEF may exert its antifungal effect by inhibiting the expression of CYP51, which in turn affects cell membrane function and leads to cells death. While FONM has a relatively high level of CYP51, rendering 0.8 mg/mL TEF not sufficient to kill the cells. This phenomenon is similar to the actions of other drugs, suggesting that TEF has the potential to act as a substitute for these drugs. However, although CYP51 is affected by TEF, there is no direct evidence to confirm that CYP51 is the direct target of TEF; instead, TEF may act directly on other sites of this pathway. In future studies, we plan to further explore changes in the levels of genes and proteins in this pathway in FONM compared to FON as well as following TEF treatment.

Notably, levels of two enzymes that could act on the carbon-carbon bond of ketonic substances were significantly reduced in FONM compared with FON. This led to the hypothesis that TEF is normally metabolized by these two enzymes to produce one or more toxic metabolites. FONM resistance may therefore result from a reduction in the level of these two enzymes. Validation of this pro-drug hypothesis will require *in vitro* drug metabolism and *in vivo* pharmacokinetic studies. Additionally, the levels of two glucosidase-related enzymes were also reduced, suggesting that the pathogenicity of FONM is weakened as a result of TEF resistance. A similar phenomenon has been reported in *Fusarium oxysporum*, wherein the pathogenicity decreased when its heat resistance increased^25^. Finally, we identified decreased expression of three proteins related to cytochrome C, which are involved the process of energy metabolism. However, this result was contrary to the higher growth rate of FONM and the underlying reason has yet to be elucidated.

## Conclusion

We have synthesized the botanical fungicide TEF and also generated a TEF-resistant mutant (FONM). *In vitro*, the antifungal effect of TEF both inhibited the growth of and directly killed FON. *In vivo*, TEF inhibited fusarium wilt in watermelons grown in the field and improved overall watermelon plant resistance. Results from proteomic and expression analyses coupled with electron microscopy imaging indicate that the cell membrane and cell wall may constitute the sites of TEF action. The mechanism of action may be related to sterol biosynthesis. Together, the findings of this study suggest that TEF may therefore represent a new botanical anti-fungicidal agent and provide the foundation for its potential development and commercial application against fusarium wilt in watermelon.

## Materials and Methods

### Materials

FON was obtained from the laboratory of plant pathology, Shanxi Agricultural University, China. TEF was synthesized by the College of Life Science, Shanxi Agricultural University, China. PDA medium was prepared according to a method described by Klepser *et al*.^26^.

FON SS was prepared as follows: FON was inoculated onto a PDA solid slant medium and then cultured for 168 h at 25 °C. FON spores were washed off with sterile water and further diluted with sterile water to yield a spore concentration of 10^6^/mL.

### GC/MS

GC/MS was used to analyse the chemically synthesized TEF using a Dexsil column (300GC 30 m × 0.25 mm × 0.25 μm, Bafang Century Technology Ltd., Beijing, China) run under the following conditions: column temperature profile, 50–250 °C at 3 °C/min; carrier gas, He; injection temperature, 250 °C; flow rate, 0.8 mL/min; injection mode, split 30:1; transfer line temperature, 290 °C; scanning range, 45–380 m/Z. Data were acquired using an online VG11-250 data system (VG Life Sciences Inc., South Pasadena, CA, USA).

### Inhibition rate and the antifungal zone of inhibition

FON SS (1 mL) was added to PDA solid medium and cultured for 168 h at 25 °C. FON were collected using a 60-mm punch and then inoculated onto PDA solid media that contained 0, 0.2, 0.4, 0.6, 0.8, or 1.0 mg/mL TEF, then incubated at 25 °C. Colony diameters were measured at 24, 48, 72, 96, 120, 144, and 168 h. The inhibition rate was calculated as (% inhibition) = (control diameter − treatment diameter) × 100/(control diameter − 60), where 60 refers to the diameter of the punch. Finally, EC_50_ values were calculated using GraphPad Prism 5.0 (GraphPad Software Inc., San Diego, CA, USA)^27^.

Different concentrations of TEF (0, 2, 4, 6, 8, or 10 mg/mL in a volume of 1 mL) were added to the centre of PDA solid medium (10 mL) containing a lawn of FON. Anti-fungal zone of inhibition circles were measured after culturing at 25 °C for 168 h.

### Morphological observation of mycelia

Slices of FON (10 mm × 10 mm × 2 mm) were obtained from FON colonies grown for 168 h. The slices were fixed using osmic acid steam (FUSHENG Biotech Co. Ltd., Shanghai, China), and observed under a JSM-7200F scanning electron microscope (JEOL Ltd., Akishima, Japan).

### FON biochemical indices

SS (1 mL) and different concentrations of TEF (0.2 and 0.4 mg/mL) were added to 100 mL of PDA liquid medium and cultured at 25 °C at 120 rpm. Samples of the culture (10 mL) were collected at 24, 48, 72, 96, 120, 144, and 168 h, and then centrifuged at 3000 *g* for 15 min. The membrane conductance and respiration intensity (i.e., the amount of CO_2_ produced) of each sample were measured according to the descriptions of Kohno *et al*.^28^ and Li *et al*.^29^.

Sodium carboxyl methyl cellulose CMC-Na (2 mL, Sigma-Aldrich Co. Ltd., Beijing, China) or salicin (2 mL, Sigma-Aldrich Co. Ltd.) was added to 0.5 mL of samples collected as above and incubated at a constant temperature of 50 °C for 30 min. The solution was inactivated by heating at 100 °C for 5 min and then cooled to room temperature. Dinitrosalicylic acid (DNS; 3 mL; Sigma-Aldrich Co. Ltd.), was added and the solution absorbance at 540 nm was measured after dilution of the sample by three fold.

### Germination rates of seed, disease index, and prevention rate

Watermelon seeds (Changle Seeds Co. Ltd., Shandong, China) were sterilized by 75% alcohol and then transferred to Petri dishes. SS (5 mL) was added followed by addition of 5 mL of sterile water, different concentrations of TEF (0.2, 0.4, 0.6, 0.8, or 1.0 mg/mL), or 0.8 mg/mL BCM (Yangnong Chemical Group Co., Ltd, Jiangsu, China). The seeds were then cultured in the absence of light at 30 °C. The number of germinating seeds was determined at 48, 72, and 96 h. Sterile water was used rather than SS as a control group. Germination rate (%) = (germinated number/total number) × 100%.

Seedlings with four true leaves were cultivated both indoor and in the field. The roots were irrigated using SS with addition of 5 mL sterile water, 0.8 mg/mL TEF, or 0.8 mg/mL BCM. The same irrigation treatment was conducted after 10 and 20 d. The treatment with sterile water was used as the control group. The disease index was assessed at 30 d and the prevention rate calculated according to previously published methods^30,31^.

### Observation of leaf ultrastructure

Seedlings with four true leaves were treated with sterile water, SS alone, or both SS and TEF (0.8 mg/mL), and then cultivated in the field. The same irrigation treatment was conducted after 24 and 48 h. After 72 h, the leaves were sliced and fixed using an osmic acid steam (FUSHENG Biotech Co. Ltd., Shanghai, China) and then observed using a JEM-100CXII transmission electron microscope (JEOL Ltd., Akishima, Japan).

### Leaf biochemical indices

Sterilized watermelon seeds were cultivated in a field until one of the following stages was attained: ‘seedlings with four true leaves’ or ‘fruiting’. SS (5 mL) was added and additional 5 mL sterile water or 0.8 mg/mL TEF was then added. Leaf tissues were collected at 24, 48, 72, 96, 120, 144, and 168 h. MDA and Pro content were measured by the thiobarbituric acid (TBA) colorimetric method^32^ and the ninhydrin coloration method^33^. SOD, POD, CAT, and PAL activity was determined according to the description of Freire *et al*.^34^; Duan *et al*.^35^; Yao *et al*.^36^, and Dong *et al*.^37^, respectively.

### Mutagenesis of FON

A diethyl sulphate (DES) solution (0.1 mL, DES:absolute ethanol, 1:4) and 8 mL of PBS pH 7.0 were added to 2 mL of SS. The mixture was incubated at 30 °C, at 120 rpm for 10, 20, 30, 40, 50, or 60 min, after which sodium thiosulfate (0.25 mL) was added. A sample (0.1 mL) of the reactant solution was inoculated onto PDA solid medium containing 0.2, 0.4, 0.6, or 0.8 mg/mL TEF. The plates were cultured at 25 °C until a single colony was established. The single colony was restreaked onto a PDA plate containing 0.8 mg/mL TEF. This process was repeated until a colony could grow in the presence of 0.8 mg/mL TEF.

### iTRAQ, RNA-Seq, and correlation analysis

SS from FON or FONM (1 mL) was added to 250 mL of PDA liquid medium and cultured at 25 °C at 120 rpm for 72 h. Total protein was extracted using the Total Protein Extraction Kit (Roche Ltd. Basel, Switzerland) according to the manufacturers’ protocols. Proteins were digested using Trypsin Gold (Promega Co., Madison, WI, USA) for 16 h at 37 °C, after which the samples were vacuum dried. The resulting peptide mixture was labelled with iTRAQ according to the manufacturer’s protocol (iTRAQ 8-plex Labeling Kit, Applied Bio, Foster City, CA, USA). The SCX fractionation and liquid chromatography-tandem mass spectrometry (LC-MS/MS) analysis were performed by Gene Denovo (Gene Denovo Bio Co. Ltd., Guangzhou, China). The primary LC-MS/MS data were converted to the MGF format using Proteome Discovery software (Thermo Scientific™, Pittsburgh, PA, USA). Protein identifications were performed using the Mascot search engine (Matrix Science, London, UK). Proteins having a significantly (*P* < 0.05) different fold change (either >1.2 or <0.83) were considered to be differentially expressed^38^. The differentially expressed proteins were analysed using GO and KEGG databases and the enrichment pathways were examined using a hypergeometric test with *P* < 0.05.

The samples for iTRAQ were also used for RNA-Seq. A Quick RNA Isolation Kit (Qiagen Co. Ltd., Suzhou, China) was used to extract total RNA. Poly(A) + mRNA was isolated using Oligo (dT) affinity chromatography. Subsequently, the mRNA was fragmented into pieces by adding a fragmentation buffer. First-strand cDNA was produced by random hexamer-primed reverse transcription, and the second-strand was generated by RNaseH and DNA polymerase I. cDNA products were purified using a QiaQuick PCR Extraction Kit (Qiagen Co. Ltd.) and dissolved in EB buffer. After end repair, the cDNA products were modified at the tail with poly(A) and Illumina paired-end sequencing adaptors. Following agarose gel electrophoresis, the cDNA fragments were extracted from gels and then enriched by PCR to construct the cDNA library. Finally, the cDNA libraries were sequenced using an Illumina HiSeq. 4000 from Gene Denovo. Sequence data were analysed and *de novo* assembly was performed using the Trinity Program. All unigenes were compared with 4 databases: Nr, COG, KEGG, and Swiss-Prot by BLASTX with cutoff E-value of 10^−5^. Significant differences in gene expression were evaluated using a threshold value of absolute log_2_-fold change >1 with a FDR <0.05 and *P* < 0.05^39,40^. The Pearson’s correlation coefficient between protein and mRNA data was calculated, and scatter plots for FON and FONM expression ratios were created.

### qRT-PCR and ELISA

SS from FON or FONM (1 mL) was added to 250 mL of PDA liquid medium with or without 0.8 mg/mL TEF, respectively, and then cultured at 25 °C at 120 rpm for 72 h. Total RNA extraction was performed using the Quick RNA Isolation Kit, and cDNA synthesis was performed using the Transcriptor First Strand cDNA Synthesis Kit (Roche Bio, Shanghai, China). Primers for qRT-PCR are listed in Supplementary Table S4 and were designed using Primer Premier 5.0 (Premier Bio, Palo Alto, CA, USA). qRT-PCR was performed using Mx3000PTM (Stratagene, Santa Clara, CA, USA) with SuperReal PreMix Plus (Tiangen Bio Co. Ltd., Beijing, China) following the manufacturer’s protocol. The relative gene expression level was calculated using the ΔΔCt method with the *TUB*2 gene as the internal control^41^.

Total protein was extracted using the Total Protein Extraction Kit (Roche Ltd., Basel, Switzerland). Standards were set up after balancing the sample concentration. ELISA was performed using a Lanosterol 14-Alpha Demethylase Kit (Multi Sciences Bio Co. Ltd., Hangzhou, China) following the manufacturer’s protocol. After the reaction was completed, the OD values of the solution at 450 nm and 570 nm were measured and the concentrations of samples were calculated.

### Statistical analysis

Statistical analyses were performed using SPSS 17.0 (IBM Co. Ltd., NY, USA). The data are expressed as the means ± standard error of mean ($\mathrm{SEM}$) and *P* < 0.05 was considered to be significant.

## Electronic supplementary material

Supplementary Information
