# Supplementary material for: Inhibitory effect and mechanism of Tagetes erecta L. fungicide on Fusarium oxysporum f. sp. niveum
Source: Sci Rep. 2017 Oct 31;7:14442. doi: 10.1038/s41598-017-14937-1 (PMC5663927; doi:10.1038/s41598-017-14937-1)
Supplement: Supplementary file 1 — Supplementary Information [file 41598_2017_14937_MOESM1_ESM.pdf]

Inhibitory effect and mechanism of *Tagetes erecta* L. fungicide on *Fusarium oxysporum* f. sp. *niveum*

Ruochen Du[1], Jiandong Liu[2], Panpan Sun[1], Hongquan Li[1*], Jinsheng Wang[2*]

[1]College of Animal Science and Veterinary Medicine, Shanxi Agriculture University, Taigu, Shanxi 030801, PR China

[2]College of Life Science, Shanxi Agriculture University, Taigu, Shanxi 030801, PR China

[*]Corresponding author(s):

Jinsheng Wang

College of Life Science, Shanxi Agriculture University, Mingxian South Road No.1, Taigu, Shanxi 030801, PR China, E-mail address: edu_sxndwjs@126.com, Tel.: +8613834834163, Fax:+86 354 6287006.

Hongquan Li

College of Animal Science and Veterinary Medicine, Shanxi Agriculture University, Taigu, Shanxi 030801, PR China, E-mail address: livets@163.com, Tel.: +8613603546480, Fax:+86 354 6288409.

SUPPLEMENTARY FIGURES

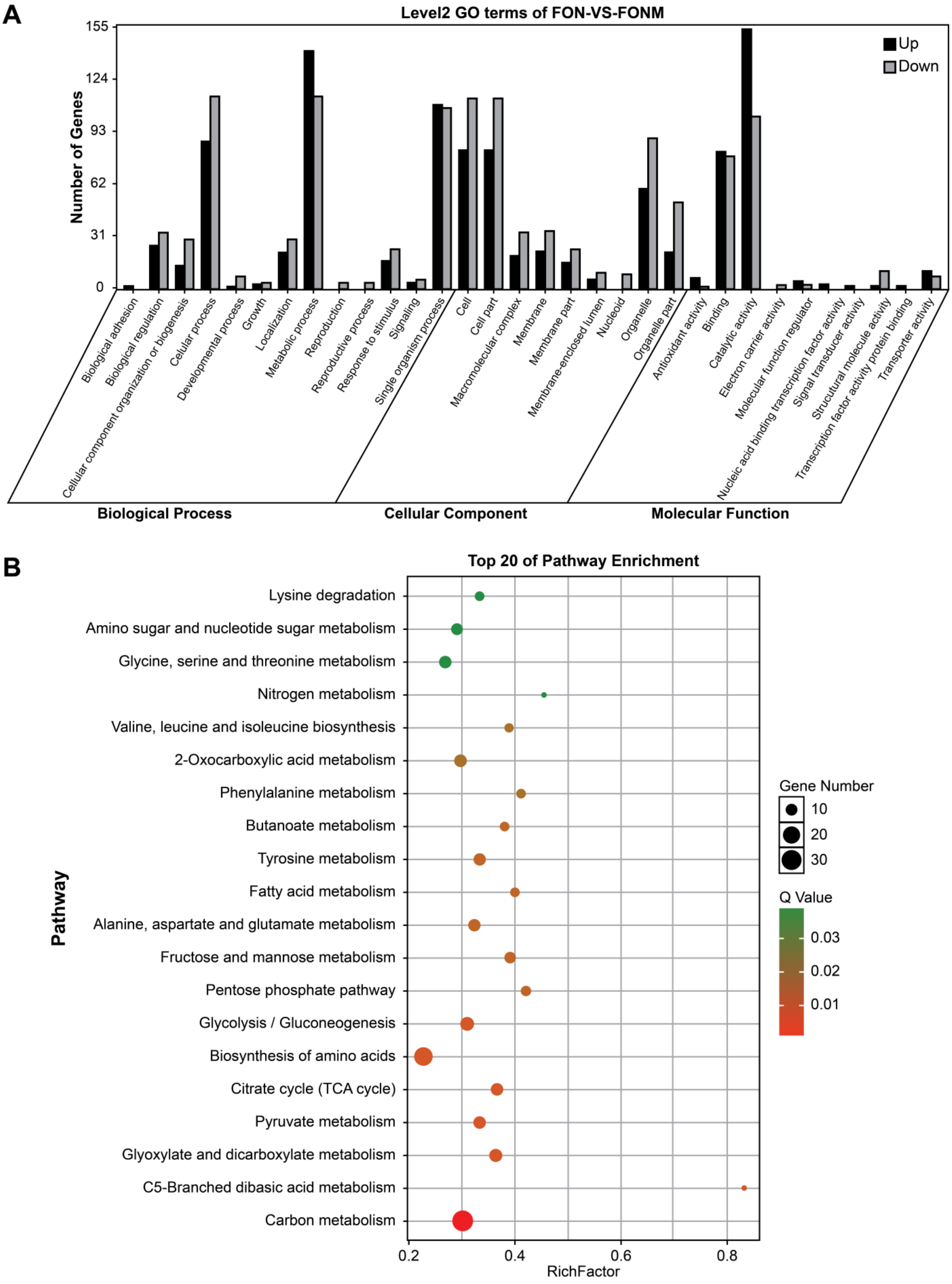

Supplementary Fig S1. GO and KEGG analysis for differentially expressed proteins

A) GO analysis

B) KEGG analysis

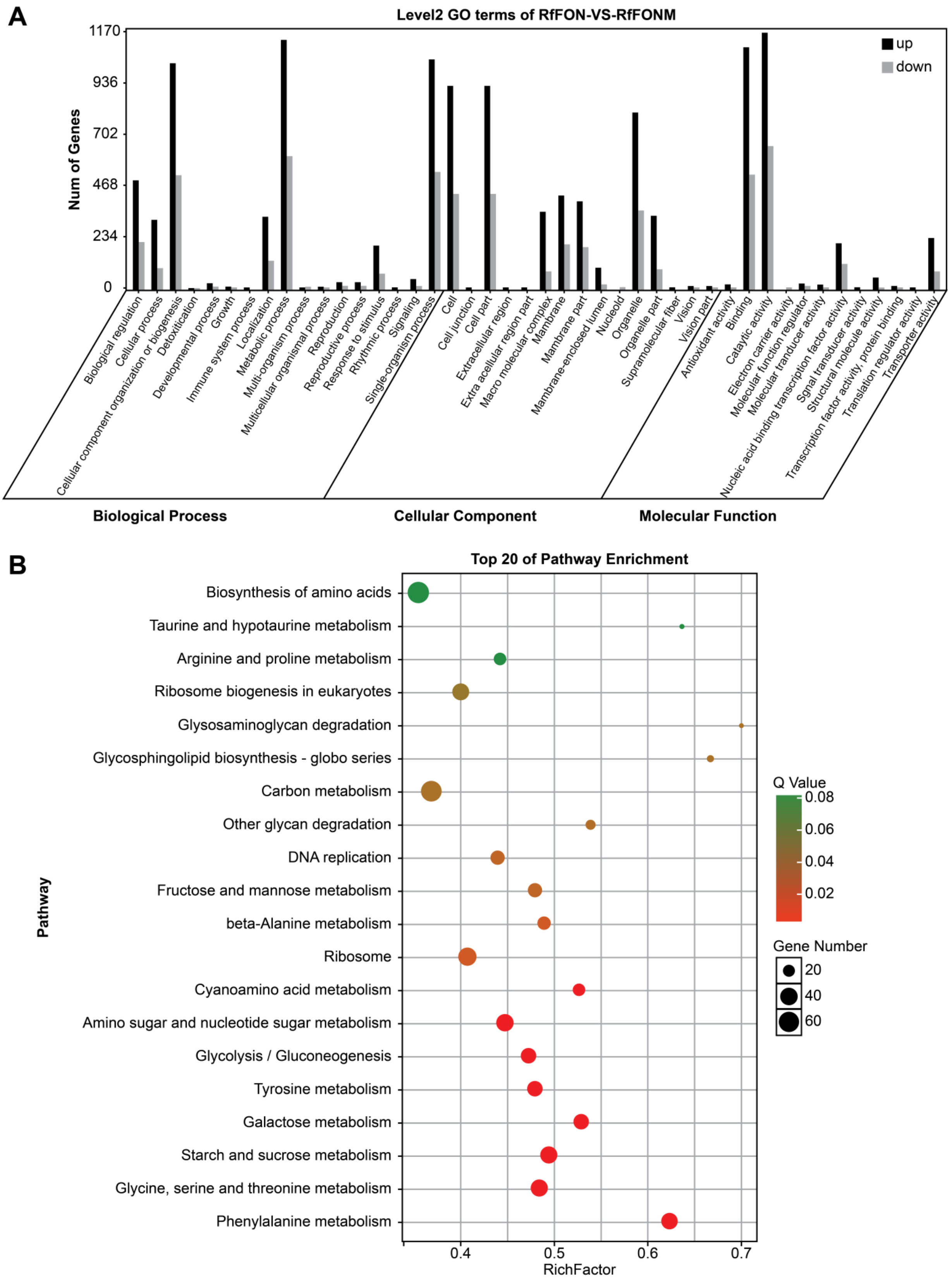

Supplementary Fig S2. GO and KEGG analysis of differentially expressed genes

A) GO analysis

B) KEGG analysis

SUPPLEMENTARY TABLES

Supplementary Table S1. Summary of iTRAQ results

Content	Number
Unique Spectra/ Spectra/Total Spectra	86157/86963/409529
Unique Peptides/Peptides	18915/19047
Proteins	3238

Supplementary Table S2. Summary of transcriptional sequencing and assembly

Database	FON	FONM
Total clean reads	132806124	108518476
Total length of clean reads (bp)	13240999690	10824084784
Q20 (%)	98.00	98.03
GC (%)	56.10	55.78
Assembly	Trinity	
Number of unigenes	20889	
Total length of unigenes (bp)	21725882	
Average unigene length (bp)	1040	
Maximum unigene length (bp)	8778	
Minimum unigene length (bp)	201	
N50 (bp)	1800	

Supplementary Table S3. The five most significant differences in protein and mRNA expression from quadrants 3 and 7

Protein Acc.	FC	Description	Organism Species	Function
		10 proteins in quadrant 3		
KPA45161.1	2.17	Methyltransferase protein	*Fusarium langsethiae*	Transferase activity
ENH63236.1	2.11	Autolysin	*Fusarium oxysporum*	-
XP_002561861.1	1.98	Pc18g00140	*Penicillium rubens*	Hydrolase activity
KPA45980.1	1.86	Leucine aminopeptidase 2	*Fusarium langsethiae*	Hydrolase and exopeptidase activity
ENH70301.1	1.81	6-hydroxy-D-nicotine oxidase	*Fusarium oxysporum*	Organic cyclic compound binding
KPA37072.1	1.72	Carboxypeptidase a4	*Fusarium langsethiae*	Metal ion binding; hydrolase activity
KPA39795.1	1.66	Membrane primary amine oxidase	*Fusarium langsethiae*	Metal ion binding; oxidoreductase activity
EWZ32741.1	1.58	Glycerol kinase	*Fusarium oxysporum*	Catalytic, kinase and transferase activity
CCT67575.1	1.49	Probable DUF895 domain Membrane protein	*Fusarium fujikuroi*	Intrinsic component of membrane
KPA38465.1	1.46	Aliphatic nitrilase	*Fusarium*	Hydrolase activity

			langsethiae	
10 RNA in quadrant 3				
KIL86238.1	9.52	Multidrug resistance protein	*Fusarium avenaceum*	Intrinsic component of membrane; Active transmembrane transporter activity
KPA38368.1	8.67	FAD containing protein	*Fusarium langsethiae*	Organic cyclic compound binding
XP_002561861.1	8.36	Pc18g00140	*Penicillium rubens*	Organic cyclic compound catabolic
EXK76462.1	8.30	Hypothetical protein FOQG_18796	*Fusarium oxysporum*	-
EXK76461.1	8.28	Hypothetical protein FOQG_18795	*Fusarium oxysporum*	-
ENH70301.1	7.52	6-hydroxy-D-nicotine oxidase	*Fusarium oxysporum*	Organic cyclic compound binding
KPA42579.1	6.50	rRNA 2-o-methyltransferase	*Fusarium langsethiae*	Intracellular organelle part
KPA38368.1	6.35	FAD containing protein	*Fusarium langsethiae*	Heterocyclic compound binding;
XP_011320935.1	6.24	Amino-acid permease inda1	*Fusarium graminearum*	Intrinsic component of membrane; Anion transmembrane transporter activity
EWG53161.1	5.94	Endoribonuclease L-PSP	*Fusarium verticillioides*	Catalytic activity
10 proteins in quadrant 7				

KPA38919.1	-1.92	22kda glycoprotein	*Fusarium langsethiae*	-
CCT63608.1	-1.73	Probable rAsp f 9 allergen	*Fusarium fujikuroi*	Hydrolase activity
KPA36188.1	-1.56	Small secreted protein	*Fusarium langsethiae*	-
KPA41277.1	-1.56	Nitric oxide dioxygenase	*Fusarium langsethiae*	Oxidoreductase activity
KPA41668.1	-1.56	Quinone oxidoreductase 2	*Fusarium langsethiae*	-
KIL85323.1	-1.05	Fumarylacetoacetase	*Fusarium avenaceum*	Hydrolase activity; acting on acid carbon-carbon bonds of ketonic substances
SCB64018.1	-0.93	Unnamed protein product	*Fusarium graminearum*	-
KPA45353.1	-0.89	Transcription factor	*Fusarium langsethiae*	-
KPA39782.1	-0.89	Phenolic acid decarboxylase PADC	*Fusarium langsethiae*	Carbon-carbon lyase activity
XP_009652260.1	-0.86	Allergen	*Verticillium dahliae*	-
		10 RNA in 7 quadrant		
KPA45183.1	-10.11	Phosphoenolpyruvate carboxykinase	*Fusarium langsethiae*	Carboxy-lyase activity

CCT64874.1	-8.15	Related to *P. aeruginosa* hyuA and hyuB	*Fusarium fujikuroi*	Catalytic activity
YP_001249308.1	-6.96	Cytochrome C oxidase subunit 2 (mitochondrion)	*Fusarium graminearum*	Energy metabolism
KPA41675.1	-5.98	DNA helicase INO80	*Fusarium langsethiae*	DNA helicase complex
KPA45353.1	-5.33	Transcription factor	*Fusarium langsethiae*	-
KPA42477.1	-5.07	DNA repair protein RAD18	*Fusarium langsethiae*	Cellular response to DNA damage stimulus
OAQ71859.1	-3.93	Helix-turn-helix domain-containing protein	*Purpureocillium lilacinum*	-
SCB64018.1	-3.87	Unnamed protein product	*Fusarium graminearum*	-
KPA46585.1	-3.41	Heat shock protein mitochondrial	*Fusarium langsethiae*	Mitochondrial part
KPA38919.1	-3.10	22kda glycoprotein	*Fusarium langsethiae*	-

FC represents Log_2(FONM/FON)

Supplementary Table S4 Sequences of primers and predicted PCR product size

Protein Acc.	Sequence (5'-3')	Product size (bp)
KPA37403.1	F:GCCTGAACACCCTCCTCTAA<br>R:TATGAAGAGAGTTGCGCCCA	133
KPA45232.1	F:TCTGTTCCCGCCATCTTC<br>R:CCGCCTTCCGTGGTCTTA	107
KPA39795.1	F:AATGGCGGGTTGTTGGTT<br>R:TCGCTTCAGTGCCTTTGC	86
CCT67575.1	F:CAGACTGGATGCCCTTGT<br>R:TCTTCTACGGTTTCTACGAT	120
EXA53151.1	F: CTCATCAGGGTTGGGGAAGT<br>R:TCCCTGGCACCAAGTATGTT	98
XP_964698.1	F:CCTGCGTAGAAGGTTGCATC<br>R:GTGGTAAACGATCCGCTGAC	169
KIL86238.1	F:GAGCTACATCTTTCGCACGG<br>R:GGACGACCGAAATGACAGTG	178
KPA44397.1	F:AAGATCCAGACCAGCGTTGA<br>R:AGAACTCTACCTCCGTCCCT	217
EXA36265.1	F:ATCTCCAAGTCTGACGCCAA<br>R:AACGTACAACTGCGCAACTT	153

KPA46791.1	F:CTCCAACGTCAACTTAGCCG<br>R:TTGTTTGAGCAGCGTCCATT	104
YP_001249308.1	F:CTACCAAATTAGTGCGAGGGG<br>R:GCTCACTTATCGGGCAAGC	224
EMT63775.1	F:TCTTCGAGGGGCTGACAATT<br>R:TGTGGAAGCCGTACCTGAAT	178
